# Transient contrast induced neurotoxicity after coronary angiography: A contrast re-challenge case

**DOI:** 10.12669/pjms.36.5.2688

**Published:** 2020

**Authors:** Muhammad Athar Sadiq, Marwa Salim Al Habsi, Sunil Kumar Nadar, Muhammad Mujtaba Shaikh, Hafidh Aqeel BaOmar

**Affiliations:** 1 Muhammad Athar Sadiq, PhD, Cardiology Unit, Department of Medicine Sultan Qaboos University Hospital Al Khoudh 123, Muscat, Oman; 2 Marwa Salim Al Habsi, MD, Cardiology Unit, Department of Medicine Sultan Qaboos University Hospital Al Khoudh 123, Muscat, Oman; 3 Sunil Kumar Nadar, FRCP, Cardiology Unit, Department of Medicine Sultan Qaboos University Hospital Al Khoudh 123, Muscat, Oman; 4 Muhammad Mujtaba Shaikh, FCPS Cardiology, Cardiology Unit, Department of Medicine Sultan Qaboos University Hospital Al Khoudh 123, Muscat, Oman; 5 Hafidh Aqeel BaOmar, FRCP, Cardiology Unit, Department of Medicine Sultan Qaboos University Hospital Al Khoudh 123, Muscat, Oman

**Keywords:** Cardiac catheterization, Contrast induced neurotoxicity, Contrast re-challenge, MRI brain

## Abstract

Contrast induced neurotoxicity (CIN) is a rare complication of cardiac catheterization and re-exposure to contrast medium carries the risk of recurrent CIN. We report a case of successful contrast re-challenge in a 60-year-old female patient who developed CIN after her first procedure of coronary angiography (CAG) which resulted in symptoms of disorientation, amnesia and cortical blindness. A non-contrast enhanced CT performed four hours after the CAG was normal, however, her MRI brain scan showed scattered tiny hyper intensities in posterior occipito-temporal and parietal regions suggesting CIN. Patient’s symptoms resolved completely after 72 hours. Two months later, because of persistent exertional angina, patient was successfully re-challenged with lesser amount of contrast medium with administration of hydrocortisone prior to procedure, and PCI to LAD was completed without recurrence of CIN.

## INTRODUCTION

Contrast induced neurotoxicity in form of fits, confusion, cortical blindness and encephalopathy is very rare complication after angiography of coronary arteries and bypass grafts.^1^,^2^ The reported incidence of contrast neurotoxicity from larger registries is 0.05% to 0.11% for diagnostic coronary angiography (CAG) and 0.3% to 0.4% for percutaneous coronary intervention (PCI).^3^-^5^ Larger volume of contrast media during cardiac catheterization is one of the risk factors associated with contrast induced neurotoxicity.^6^ Re-exposure of the iodinated contrast media during subsequent cardiac catheterization carries the risk of recurrent contrast induced neurotoxicity.^7^,^8^ We identified only three cases of cortical blindness after cardiac catheterization where contrast media was successfully re-challanged.^9^ Here, we report a case of successful re-challenge of contrast medium to a post-CAG contrast induced neurotoxicity patient and purpose a criteria for contrast medium re-challenge.

## CASE REPORT

**Fig.1:**
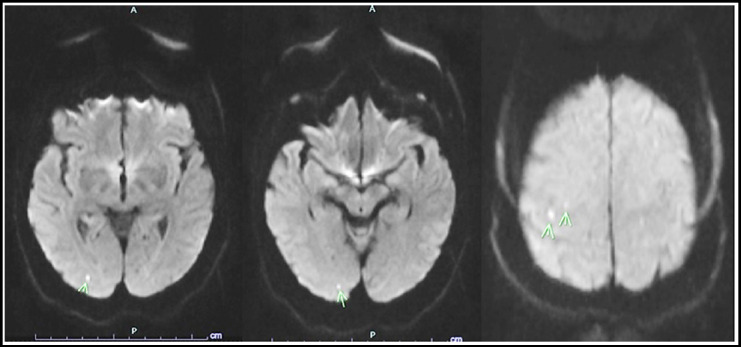
Brain MRI images showed tiny scattered hyper intense areas (arrows) in the right posterior occipito-temporal and parietal lobes.

A-60-year old woman with history of hypertension, dyslipidemia and paroxysmal AF admitted for acute coronary syndrome and underwent CAG. Her CAG was performed through right radial access with 5F Judkins diagnostic catheters. There was significant tortuosity in her brachiocephalic trunk which resulted in difficult engagement of both left and right coronary systems, hence larger volume (300 ml) of contrast media (Iohexol) was administered during her coronary angiography. Patient did not have a previous history of any procedure with contrast agent exposure. Her CAG showed significant 70% & 90% tubular lesions in proximal and mid segments (respectively) of left anterior descending coronary artery (LAD) requiring intervention. However, patient started to become confused, aggressive and later developed amnesia and cortical blindness. Her neurological examination did not reveal any cranial, sensory or motor nerves abnormality. A non-contrast computerized tomography (CT) scan performed four hours after the start of symptoms showed no acute pathological findings. Her brain magnetic resonance imaging (MRI) demonstrated tiny hyperdensities in the posterior occipito-temporal and parietal regions suggesting contrast induced neurotoxicity (Fig.1). Patient’s electroencephalogram (EEG) showed no epileptiform discharge. She was managed conservatively with intravenous fluids, and received haloperidol and midazolam when needed. A total recovery was noted 72 hours after the onset of symptoms. After 2 months, patient was electively admitted for PCI to her LAD for having persistent exertional angina symptoms. Prior to PCI procedure, she was given a single dose of 200 mg of intravenous hydrocortisone in cardiac catheterization lab and right femoral access was obtained to minimize the amount of contrast. She underwent successful PCI to both of her LAD lesions after a safely re-challenge of 60 ml of Iohexol contrast agent. She was discharged one-day post PCI without recurrence of any of symptoms of contrast neurotoxicity.

## DISCUSSION

Contrast induced neurotoxicity (CIN) following cardiac catheterization is a rare but devastating complication. Its clinical presentation varies from mild symptoms of headache and vomiting to more serious presentation with seizures, hemiparesis, encephalopathy and cortical blindness.^7^,^8^ Cortical blindness is the most common presentation of contrast induced neurotoxicity, and is present in more than 50% of cases.^9^ CIN is an acute reversible neurological disturbance, fully resolving within 48-72 hours with a benign outcome but may result in persistent neurological deficit or death.^10^,^11^ Brain imaging by CT or MRI plays an important role in differentiating CIN from other neurological conditions and thromboembolic complications associated with cardiac catheterization. CT findings vary from normal to cortical/subcortical contrast enhancement, bilateral subarachnoid hyperdensities and cerebral edema.^6^,^12^ MRI scans can reliably differentiate CIN form cerebral ischemia. In our patient a non-contrast CT scan performed four hours after the start of symptoms showed no acute pathological findings. However, her brain MRI demonstrated very tiny multiple hyper densities in the right posterior occipito-temporal and parietal regions suggesting contrast induced neurotoxicity (Fig.1).

The exact mechanism of contrast induced CIN remains unknown. Disruption of blood brain barrier and direct contrast neuronal toxicity mainly in the occipital region of brain haven been postulated as a possible mechanism in many reports.^2^ All kind of iodinated contrast media, irrespective of their osmolarity or isonic state can result in contrast induced neurotoxicity. Contrast media re-exposure to patients with history of CIN after cardiac catheterization carries the risk of recurrent CIN. Law et al. reported a case of contrast induced encephalopathy, following administration of iodixanol, which resolved completely within 24 hours. It was subsequently determined that one year earlier, patient had experienced transient binocular blurred vision following a cardiac catheterization in other hospital.^7^ Spina et al. described a case of recurrent contrast induced encephalopathy after cardiac catheterization two years apart.^8^

Contrast media re-challenge has not been widely reported in literature. We identified only three cases where re-challenge of contrast media did not result in recurrence of cortical blindness.^9^ Role of pre-medication with intravenous corticosteroids in preventing CIN is uncertain. In a case report described by Spina et al., pre-medication with intravenous corticosteroid did not prevent CIN on first occasion and recurrence of CIN during second cardiac catheterization procedure. Administration of large volume of contrast media during cardiac catheterization is an established risk factor for CIN.^1^ Although the maximal recommended dose for coronary angiography to prevent CIN is not known, some studied have proposed 170 ml to 200 ml as the maximum recommended dose.^1^, ^6^ In our patient, during her first coronary angiography procedure which was performed through right radial access, large volume of 300 ml of Iohexol was used due to significant tortuosity in her brachiocephalic trunk which resulted in difficult engagement of both left and right coronary systems. During her second procedure, right femoral access was obtained to minimize the amount of contrast medium and successful PCI to LAD was completed with 60 ml of contrast volume. Similar approach was adapted by Rama et al. in a CIN patient who was successfully re-challenged by minimizing the amount contrast dye and pre-treatment with intravenous carticosteriods.^9^

Herein, we propose that re-challenge with minimal amount of contrast medium after pre-treatment with intravenous corticosteroids can be considered in patients with previous history CIN if: i) a second procedure is mandated as was the case in our patient to have persistent angina despite optimal anti-anginal medical therapy ii) CIN resolves completely after the first contrast exposure and iii) larger volume of contrast is associated with CIN.

## CONCLUSION

CIN following coronary angiography is an extremely rare but usually reversible complication. Re-exposure of contrast media to patients with history of CIN carries the risk of recurrent CIN, hence it is not well documented. We successfully re-challenged contrast medium in our patient and proposed that contrast rechallenge may be considered in some patients if certain conditions are fulfilled However, it is difficult to conclude whether or not CIN will recur with contrast re-challenge but we are reassured with the fact that CIN is usually transient and resolves completely.

### Author`s Contribution

**MAS & MWA** did manuscript writing. **MMS** edited the manuscript

**SKN** reviewed the manuscript. **HAB** gave final approval. **MAS** responsible and accountable for the accuracy or integrity of this study.
